# Development of a Low-Cost Infrared Imaging System for Real-Time Analysis and Machine Learning-Based Monitoring of GMAW

**DOI:** 10.3390/s25226858

**Published:** 2025-11-10

**Authors:** Jairo José Muñoz Chávez, Margareth Nascimento de Souza Lira, Gerardo Antonio Idrobo Pizo, João da Cruz Payão Filho, Sadek Crisostomo Absi Alfaro, José Maurício Santos Torres da Motta

**Affiliations:** 1 Programa de Engenharia Metalúrgica e de Materiais, Universidade Federal do Rio de Janeiro (UFRJ), Rio de Janeiro CEP 21941-972, RJ, Brazil; jairojmch@metalmat.ufrj.br (J.J.M.C.); jpayao@metalmat.ufrj.br (J.d.C.P.F.); 2 Programa de Pós-Graduação em Engenharia Mecatrônica, Departamento de Engenharia Mecânica, Universidade de Brasília (UnB), Brasília CEP 70910-900, DF, Brazil; gerardo_idrobo@unb.br (G.A.I.P.); sadek@unb.br (S.C.A.A.); jmmotta@unb.br (J.M.S.T.d.M.)

**Keywords:** infrared imaging, GMAW monitoring, real-time image processing, low-cost sensors, weld bead geometry

## Abstract

This research presents a novel, low-cost optical acquisition system based on infrared imaging for real-time weld bead geometry monitoring in Gas Metal Arc Welding (GMAW). The system uniquely employs a commercial CCD camera (1000–1150 nm) with tailored filters and lenses to isolate molten pool thermal radiation while mitigating arc interference. A single camera and a mirror-based setup simultaneously capture weld bead width and reinforcement. Acquired images are processed in real time (10 ms intervals) using MATLAB R2016b algorithms for edge segmentation and geometric parameter extraction. Dimensional accuracy under different welding parameters was ensured through camera calibration modeling. Validation across 35 experimental trials (over 6000 datapoints) using laser profilometry and manual measurements showed errors below 1%. The resulting dataset successfully trained a Support Vector Machine, highlighting the system’s potential for smart manufacturing and predictive modeling. This study demonstrates the viability of high-precision, low-cost weld monitoring for enhanced real-time control and automation in welding applications.

## 1. Introduction

Welding is one of the most widely used manufacturing processes in modern industry, and welding has been extended to the field of additive manufacturing with wires and arcs (also known as additive welding), making research into monitoring and controlling printing process parameters essential for the evolution of this field of study that has emerged in recent decades [1,2,3,4]. The quality assurance of welded joints and components produced through additive manufacturing using welding relies on non-destructive and destructive testing methods. Non-destructive testing, such as visual inspection, ultrasonic testing, magnetic particle inspection, and liquid penetrant testing, detects surface and subsurface discontinuities without compromising the part’s integrity [5,6,7]. In contrast, destructive testing is employed to evaluate key properties, such as mechanical strength, corrosion resistance, and microstructural characteristics [8,9]. Typically performed after welding, these inspections may cover the entire weld length depending on the application [10,11].

In contrast, real-time monitoring during welding enables the immediate evaluation of parameters such as bead geometry, arc length, and process stability. Studies by Xu et al. [9], Mishra et al. [12], and Sarkar et al. [13] have demonstrated that computer vision systems, when integrated with signal processing algorithms, can dynamically adjust electrical and mechanical parameters to improve weld quality. The use of infrared sensors in such vision systems for fusion welding has been investigated by Naksuk et al. [14], Hamzeh et al. [15], Wang et al. [16], and Khan et al. [17], enabling the detection of temperature variations and differences in metal emissivity. These variations, in turn, facilitate image segmentation and the extraction of geometric features from the weld bead.

Li et al. [18] developed an automated line scan profilometer based on the surface recognition method. The experimental measurements validate the advantages of this method over existing methods in terms of measurement efficiency, automation capability, and applicability. As noted by Hou et al. [5], computer vision systems in welding can be applied to process inspection, parameter monitoring, trajectory correction, and the analysis of transient physical phenomena such as heat flow, metal phase transitions, and droplet formation. However, the intense and multispectral radiation emitted by the electric arc presents challenges for infrared imaging. Wang [19] controlled the welding process by monitoring the weld pool with reflected laser lines, which are projected onto the specular weld pool and reflected onto a diffusive plane.

Chokkalingham et al. [20] estimated the weld bead width and penetration depth from real-time infrared images. However, at higher current values, the maximum temperature exceeds the measurement range of the infrared (IR) camera, rendering the input peak temperature values meaningless. To tackle the problem of meaningless peak temperatures under high-current welding conditions, where temperatures exceed the measurement range of the IR camera, Wang et al. [16] used the thermal area under the transverse profile of the temperature distribution at the weld pool width position instead of peak temperature.

In accordance with Santoro et al. [21], thermography possesses its unique set of advantages and disadvantages. On the one hand, the non-invasive nature of thermography, coupled with its capacity to provide real-time, continuous monitoring of a process, renders it particularly suitable for applications such as welding. On the contrary, the reliability of thermography can be influenced by variables such as ambient temperature and material emissivity, potentially compromising the accuracy of readings [22]. Furthermore, the interpretation of thermographic data can prove to be complex, necessitating the use of sophisticated software and skilled operators.

These limitations are further exacerbated by the welding process’s inherently chaotic nature, which involves many interacting variables. In such a system, even a slight variation in one parameter can lead to significant changes in others, thereby increasing the complexity and the computational cost of real-time control algorithms. Moreover, the high cost of commercial systems restricts their accessibility. Furthermore, the developed infrared cameras were designed to detect hot bodies [23] at various temperatures, ranging from the body temperature of living beings to high-temperature ovens. Each of these situations presents an ideal electromagnetic range [24].

Industrial cameras used for welding (e.g., Optris, NIT, Xiris) are still in constant development and do not have a clear range for welding applications. This is because these cameras were not initially focused on welding, which does not take into account the specificities of plasma with different gases. To address these challenges, this study proposes a low-cost (up to 45 times cheaper than solutions currently offered on the market) optical acquisition system based on infrared cameras for real-time measurement of weld bead geometry during Gas Metal Arc Welding (GMAW), to achieve a measurement error below 1%. The focus of this work is the study by spectroscopy of the ideal electromagnetic bands that an infrared camera should have in welding and their change in the different elements that make up the plasma. Unlike commercial cameras, the primary difference lies in the use of appropriate filters rather than a high-cost sensor with high resolution. Understanding how to apply specific filters for each type of plasma optimizes both costs and the quality of filming the molten pool, thereby extending the use of the technique to various materials and shielding gases involved in welding processes.

## 2. Materials and Methods

This study employed an experimental methodology using low-cost commercial optical cameras and filters and custom-designed image processing algorithms. These algorithms were developed based on blackbody radiation principles and spectral analysis of gas and metal emissions. Weld bead depositions were monitored via automatic profilometry, utilizing magnetic sensors and 3D digitizers, achieving a measurement accuracy within 1%.

A computer vision system was implemented to visualize weld bead geometry in real time during the GMAW process. The system utilized a commercial CCD camera sensitive (Model CAM-1080p Full HD 30 FPS, Intelbras Inc, São José, Brazil) to wavelengths from 350 nm to 1150 nm, along with optical components like lenses, attenuators, and filters optimized for the 1000–1150 nm infrared range.

**Figure 1:**
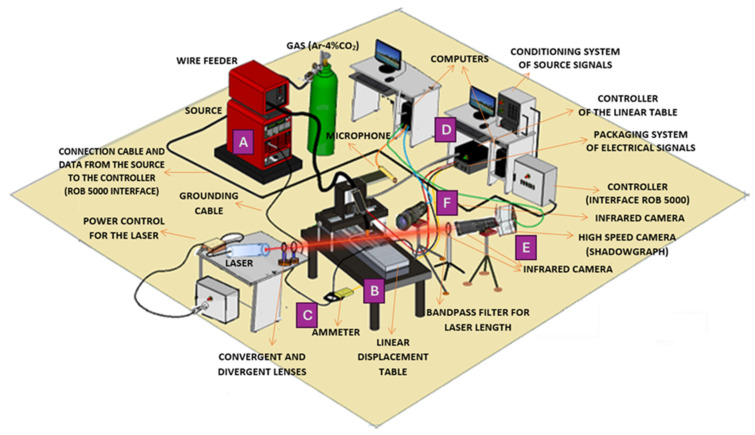
Experimental workbench showing the monitoring system and welding equipment. Where (A) power source; (B) linear displacement table; (C) ammeter and voltmeter; (D) data acquisition system; (E) CCD camera; (F) high speed infrared camera.

The experimental setup, shown in Figure 1, was structured into three main modules:

Module I—Welding Parameter Control

(A) Power source (Model TransPlus Synergic 5000, Fronius, Inc., Pettenbach, Austria): the power source was configured to operate with a constant voltage output and variable wire feed speed control, specifically optimized to meet the requirements of the Gas Metal Arc Welding (GMAW) process.
(B) Linear displacement table: This custom-built mechanical system provided controlled linear motion for both the workpiece and the welding torch. This setup enabled precise adjustments of travel speed and vertical positioning during welding.

Module II—Sensing of Electrical Parameters

(C) Ammeter (Model i1010 AC/DC, Fluke Corporation Inc., Everett, WA, USA) and voltmeter (Model DVL 500, LEM Inc., Geneva, Switzerland): these instruments were used to monitor key welding parameters in real time, including the welding current near the arc and the voltage potential difference between the welding torch and the base material.
(D) Data acquisition system (Model PCI Eagle 703s, Eagle Technology, Inc., Cape Town, South Africa): A high-speed data acquisition board was used to collect electrical signals (current and voltage) generated during welding. The acquired data were processed and analyzed using the LabVIEW and MATLAB R2016b software platforms.

Module III—Computer Vision and Results Validation

(E) CCD camera: a commercial CCD camera, sensitive to a broad spectral range (350–1150 nm), was employed and operated explicitly in the near-infrared range (1000–1150 nm) to capture real-time images of the evolving weld bead.
(F) High-speed infrared camera: This specialized camera captured rapid sequences of thermal images during welding, enabling detailed analysis of phenomena such as molten droplet formation, detachment from the electrode, and the thermal profile of the weld pool and surrounding material. A high-speed camera (Model 1M150-SA, Teledyne DALSA Inc., Waterloo, ON, Canada) with monochrome CMOS technology was used, offering 256 gray levels and a resolution of 96 × 128 pixels, with an acquisition rate of 1000 fps and a CMOS sensor exposure time of 50 µs. The light source was a He-Ne laser (633 nm, 15 mW) (Model He-Ne 633 nm and 15mW, Excelitas Technologies Inc., Waltham, MA, USA). To maintain a constant beam radius, a Galilean beam expander was constructed using a diverging lens (focal length = 40 mm) and a second converging lens.
(G) Laser profilometer and optical filters: A laser profilometer (Model LLT 2950-100/BL, Micro-Epsilon Messtechnik GmbH & Co. KG Inc., Ortenburg, Germany) was used to perform precise geometric measurements of the solidified weld beads in conjunction with specific optical filters. The filters enhanced the clarity of the projected laser line and isolated its specific wavelength, enabling accurate measurement through point cloud data analysis. Each component is labeled in Figure 1 to facilitate correlation between the experimental setup and the visual representation.

In Modules I and II, the welding process was performed using a Fronius TransPlus Synergic 5000 power source operating at constant voltage with variable wire feed speed. A shielding gas mixture of 96% Ar and 4% CO_2_ was applied at a flow rate of 15 L/min. Electrical signals (current and voltage) were acquired near the welding arc and between the torch and the base plate using a PCI Eagle 703s data acquisition card. Signal processing was conducted using the LabVIEW and MATLAB^®^ platforms.

The materials we used included the following:

- Filler metals: AISI ER316L solid wire (1.2 mm diameter) and E410NiMoT1-4 tubular wire (1.2 mm diameter) from ESAB Inc., North Bethesda, MD, USA. The chemical composition is shown in Table 1.
- Substrate: AISI 1020 (Aperam South America Inc., Timótio, Brazil) steel plates (6.35 mm × 250 mm × 50 mm).

A linear displacement table controlled the precise positioning and movement of the workpiece and welding torch. Electrical parameters (current and voltage) were measured near the arc and at specific torch and table surface points using a voltmeter configured as a voltage divider.

In Module III, additional instrumentation was employed:

- An infrared camera assessed weld bead length, reinforcement, and width.
- A laser light and high-speed camera was utilized for droplet analysis via the shadowgraph technique.
- Optical filters and lenses were employed to isolate relevant spectral bands and enhance image contrast.
- A laser profilometer was used to capture weld geometry through point cloud reconstruction.

Two primary methodologies were applied for data collection:

1. Parametric sweep: systematic voltage and wire feed speed variation while maintaining other parameters at fixed values.
2. Full factorial variation: simultaneous voltage, wire speed, and welding speed variation.

The experimental design followed a central composite design (CCD) approach, encompassing 35 experimental runs to validate the system’s accuracy and robustness.

Module IV consisted of the integration of experimental data obtained online with a machine learning algorithm based on a Support Vector Machine (SVM).

## 3. Results and Discussion

### 3.1. Optical Analysis Through the Electromagnetic Spectrum

The optical analysis in this study was conducted in two distinct stages, each focusing on a specific region of the electromagnetic spectrum: 60–810 nm and 810–1200 nm. This division was necessitated by the spectrometer’s technical limitations, which had a detection limit of approximately 810 nm.

**Figure 2:**
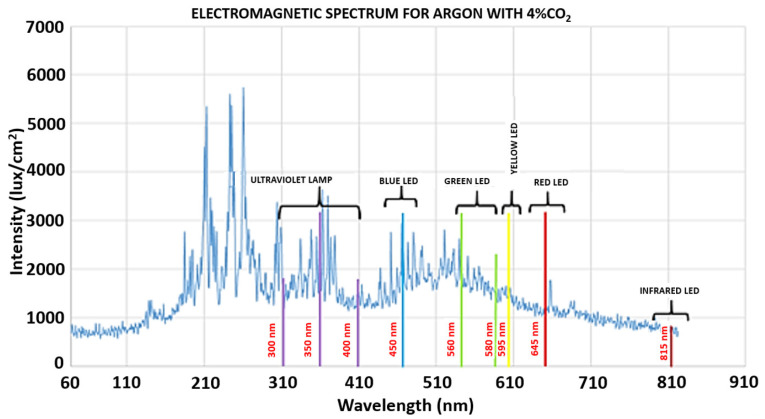
Emission spectrum of argon with 4% CO_2_ during GMAW, with superimposed spectra from LEDs and an ultraviolet lamp for peak identification.

Figure 2 illustrates the electromagnetic spectrum of argon mixed with 4% CO_2_ during the GMAW process. Known emission spectra from ultraviolet lamps and commercial LEDs were superimposed to help identify characteristic peaks. Table 2 presents the typical emission wavelength ranges associated with commercial LED colors to support this analysis.

The results in Figure 2 indicate that the light intensity emitted by the electric arc can exceed 1000 lux/cm^2^, approaching the saturation limit of typical commercial imaging sensors. This saturation impedes accurate visualization of the weld bead geometry. Prior studies, such as Wang et al. [25], have mitigated this issue using filters, attenuators, and high-power external infrared illumination, often in the 750–850 nm range, to reduce image saturation. However, these additions increase the setup’s complexity and cost. In contrast, a key objective of this study is to eliminate the requirement for external lighting, thus reducing equipment costs and simplifying implementation.

The second spectral stage, spanning 810 to 1200 nm, was analyzed in comparison to previous findings by Mota et al. [26] and Zhang et al. [27]. Mota et al. [26] identified firm radiation peaks within the 896–922 nm range, consistent with blackbody radiation characteristics. These high-intensity peaks were found to compromise image quality. However, in spectral regions beyond 923 nm, image capture tends to be more effective due to the lower presence of gas radiation peaks, which are associated with temperature increase and electronic transport phenomena in the plasma, thereby improving the visualization of the weld bead geometry in the infrared camera.

Zhang et al. [27] further expanded this analysis in their study of the GTAW with argon shielding gas. Their findings confirmed additional radiation peaks below 850 nm and within the 890–922 nm band, contributing to sensor saturation. Conversely, the 850–896 nm and 923–1100 nm intervals were identified as favorable imaging windows due to lower radiation density.

**Figure 3:**
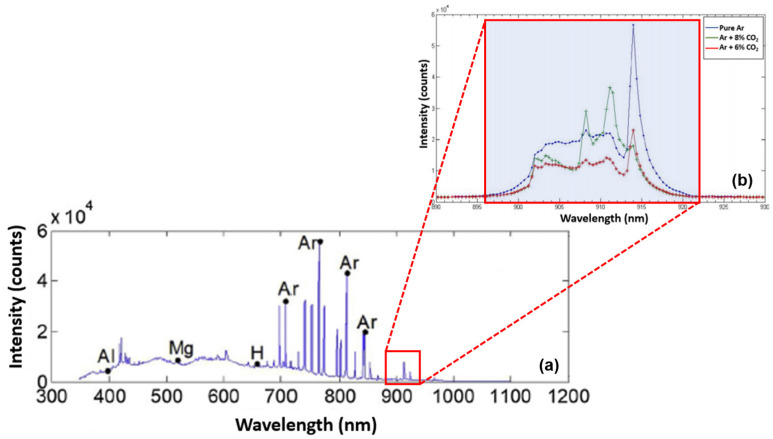
Electromagnetic spectra for argon: (**a**) from 300 to 1200 nm and (**b**) magnified view of the 890–930 nm range. Adapted from Mota et al. [26] and Zhang et al. [27].

Figure 3a presents the complete electromagnetic spectrum of argon from 300 to 1200 nm, while Figure 3b provides a zoomed-in view of the 890–930 nm range. Based on pure argon and argon mixtures with 5% and 8% CO_2_, this comparison illustrates that the intensity peaks most significantly impact image acquisition.

Based on these findings, the 805–923 nm range presents significant challenges for accurate imaging due to high spectral interference. Consequently, this work emphasizes using wavelengths above 920 nm, particularly within the 1000–1150 nm interval, as these provide more precise visual data with minimal interference. This approach enhances real-time analysis of weld bead geometry without the need for costly active illumination systems.

The upper limit of 1100 nm was determined by the response range of the CCD camera, which exhibits reduced sensitivity beyond this point. Therefore, the selected range represents an effective balance between spectral clarity and sensor performance, further supporting the feasibility of low-cost optical monitoring systems in welding.

### 3.2. Development of Vision Systems

**Figure 4:**
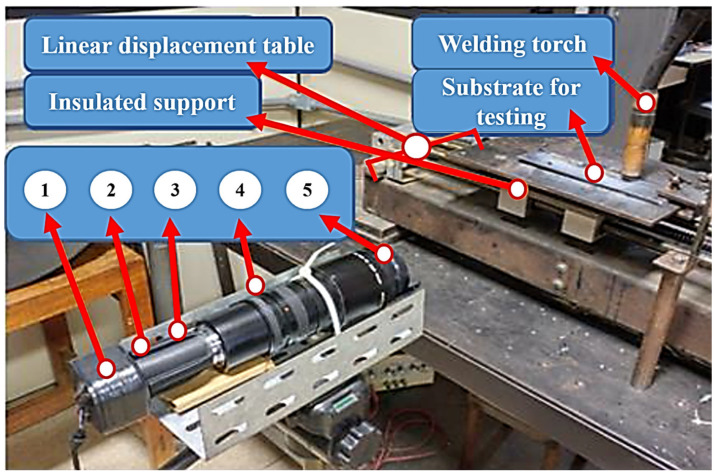
Experimental camera and lens assembly, where (1) a 2.0-megapixel webcam operating at 30 to 50 fps, adapted for infrared; (2) 1000 nm long-pass infrared filter lenses; (3) two 1.0 optical density radiation attenuators; (4) a telephoto zoom lens with a focal length range of 18–108 mm and aperture of 2.5; and (5) a polarizer were used.

Building upon the optimal spectral range for weld bead observation identified in Section 3.1 (above 920 nm), we designed and implemented a computer vision system tailored to this spectral window (Figure 4). The objective was to capture thermal emissions from the weld pool while minimizing the interference from intense visible electric arc radiation.

#### 3.2.1. Camera and Optical Assembly

The selected configuration comprised a 2.0-megapixel CMOS webcam adapted for infrared detection (Figure 4(1)), with a sensitivity range extending to 1150 ± 50 nm. Combined with a 1000 nm long-pass filter (Figure 4(2)) and two 0.1 ND attenuators (Figure 4(3)), this setup effectively filtered out visible light and plasma glare. A telephoto zoom lens (18–108 mm, aperture 2.5) (Figure 4(4)) and a polarizing filter (Figure 4(5)) were integrated to enhance image contrast and provide system protection.

**Figure 5:**
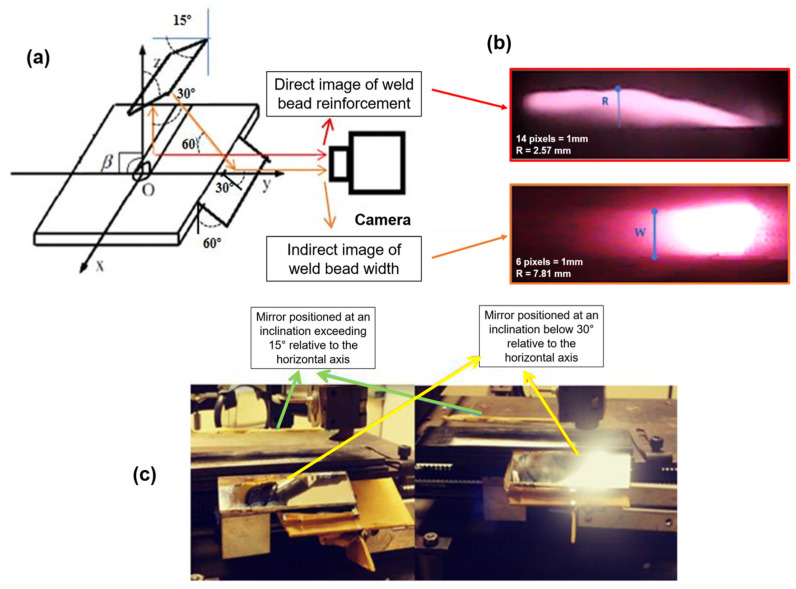
Observation system for weld bead geometry measurement, including pixel-to-millimeter scaling. (**a**) Schematic of mirror and camera positioning. (**b**) Image acquisition using the infrared camera. (**c**) Positioning of the horizontal and vertical mirrors in the experimental setup. Where the arrows red indicates the path for measuring the reinforcement; the orange indicates the path for measuring the width of the bead; the blue indicates the width and reinforcement dimensions; the yellow indicates the mirror positioned at 30° in relation to the horizontal axis; and the green indicates mirror positioned at 15° in relation to the horizontal axis.

A 180° mirror-based observation system was constructed, as illustrated in Figure 5, to enable the simultaneous acquisition of weld bead reinforcement and width measurements. This setup allowed for indirect visualization of the weld bead from two distinct perspectives using a single camera. The system was calibrated using the RAC algorithm [28] and reference objects, including infrared LEDs. In the used experimental setup, the mirrors, torch, and camera are kept stationary, while the welding table is responsible for the travel speed. The table features a single degree of freedom and operates as a linear motion system along a rail.

**Figure 6:**
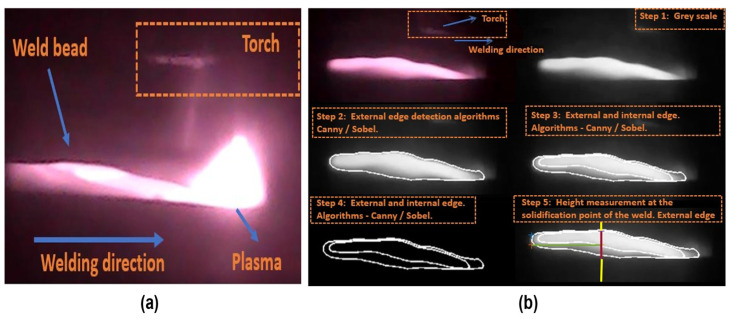
(**a**) Unprocessed IR image of the weld bead. (**b**) Stages of the edge detection algorithm. The yellow arrow indicates the substrate reference point, and the red arrow indicates the reinforcement measurement provided by the segmentation algorithm.

Following image capture, real-time image processing algorithms were applied to extract weld bead geometric parameters. Figure 6a shows a raw IR image, while Figure 6b displays the segmented edges obtained using a combination of Canny, Sobel, and Prewitt filters.

The developed equipment captures images of the weld bead online during the welding process. These images enable viewing the arc length and the geometry of the bead forming near the weld pool. These images are not affected by the intense light of the arc. Another critical point is the possibility of viewing both the bead and the arc length simultaneously. A positive point is the fact that the photons generated by the thermal radiation of metals (black body radiation) in this wavelength range have a greater intensity, while that generated by the plasma decreases, thus balancing the radiation of the metal with the radiation generated by the arc.

Plasma has a lower radiation intensity in this wavelength range, in addition to having a lower density than metal, so the total contribution of radiation intensity when considering the sum of each atom is lower. This measurement of radiation intensity is linked to the excitation of each atom by the increase in temperature or vibration that generates a quantity of photons in the infrared range. Therefore, the total intensity in the study range decreases for plasma and increases for metal.

Although the metal has a higher density, which favors greater radiation, the gas or plasma has a higher temperature compared to the molten metal, causing a balance in intensity. Another advantage of viewing the bead with the infrared camera is that the temperature of the metal dissipates more slowly than that of the gas, allowing the image to be viewed for a more extended period, even if the arc moves away or is extinguished. Therefore, the intensity of the radiation due to the increase in temperature of the metal and plasma is the key to better viewing, allowing a wavelength range to be established where the intensity generated by the plasma is lower and that of the metal is higher, thereby balancing them to enable viewing of the bead.

In addition to choosing filters that work well for the application in question, all images showed a lot of brightness and light saturation from the electric arc. To overcome this problem, the use of two attenuators in the optical system was crucial for obtaining greater definition at the edges and sharper weld beads. The adjustment of the attenuators and the filter range between 950 and 1050 nm must be made for each change of wire and gas. This is a simple calibration, but it ensures the system’s use in a generalized manner rather than just for a specific class of material or gas.

**Figure 7:**
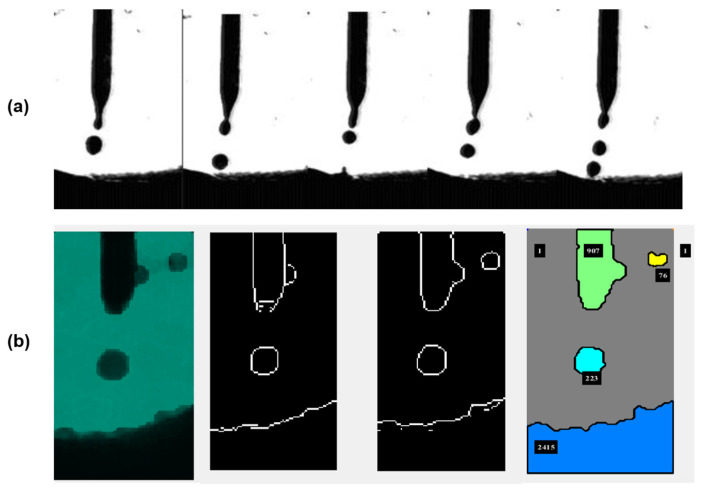
(**a**) Sequences of images from the high-speed camera with droplet mode configuration. (**b**) Image processing by edge surface with object recognition and pixel calculation, where each color is randomly assigned by the algorithm to represent a different pixel intensity.

The high-speed camera uses the profilography technique to acquire images of the metal transfer mode at an acquisition rate of 800 frames per second. The obtained images are synchronized with the electrical signals and the images obtained with the infrared camera, making it possible to correlate them with the instantaneous values of welding current and voltage. The frequency of 800 frames per second enables the visualization of drop formation and detachment while also preventing the saturation of computational resources within a few seconds. Thus, it is possible to visualize the changes in the formation and detachment of the drop in the different transfer modes: short circuit, globular, and spray. Figure 7a shows a sequence of images taken with the high-speed camera in the droplet transfer mode, and Figure 7b shows the processing of images by edge surfaces with object recognition and pixel calculation.

#### 3.2.2. Image Processing Algorithm

The data analysis was performed using MATLAB R2016b (The MathWorks, Inc., Natick, MA, USA). Each step of the algorithm is described in brief in Appendix A.

The algorithm processes each frame as follows:

Step 1—load and preprocess the image:

Frames are loaded sequentially. A median filter is applied for noise reduction, and the image is then converted to grayscale (rgb2gray) for subsequent edge processing.

Step 2—external edge detection:

A binarization threshold (Gray ≤ 100) isolates the hotter outer boundary of the weld. Edge detection filters (Sobel, Canny, Laplacian of Gaussian (LOG), Prewitt) are then applied to this binary image.

Step 3—internal edge detection:

A second threshold (Gray ≤ 200) targets the internal region of the weld bead. The same edge detection filters are applied, resulting in the internal edge map (EdgeF2).

Step 4—calculation of the column on the X-axis, where the weld bead reinforcement should be measured:

A central value on the X-coordinate, denoted as Xm, corresponding to the average between the outer and inner edges, is calculated on the side opposite to the plasma position. This value is slightly shifted toward the plasma by multiplying it by 0.95, which reduces the distance between the plasma center and the end of the weld pool. This adjusted position is defined as the variable XR, representing an X-axis location in the image that is ideal for measuring the reinforcement or weld bead height shortly after solidification.

For cases where the plasma image is not well defined or its center cannot be located, or where the inner and outer edges present errors, a second method should be used. This method consists of an equation developed through regression that calculates the point where the reinforcement should be measured on the X-axis, based on the values of Ws and voltage. The development of this equation is presented in Section 3.2.3.

Step 5—measurement of the weld bead reinforcement (Figure 7):

The pixel values are continuously measured by scanning along the XR column, identifying pixels with a value of 1 and their position (X, Y) based on the image edges, which were created during the image processing step. In this way, the lower and upper values for each column and each edge are collected, with the measurement being taken from the top (Start_Reinforcement) to the base (End_Reinforcement). The difference in the pixel row numbers between these two points produces the reinforcement in pixels.

**Figure 8:**
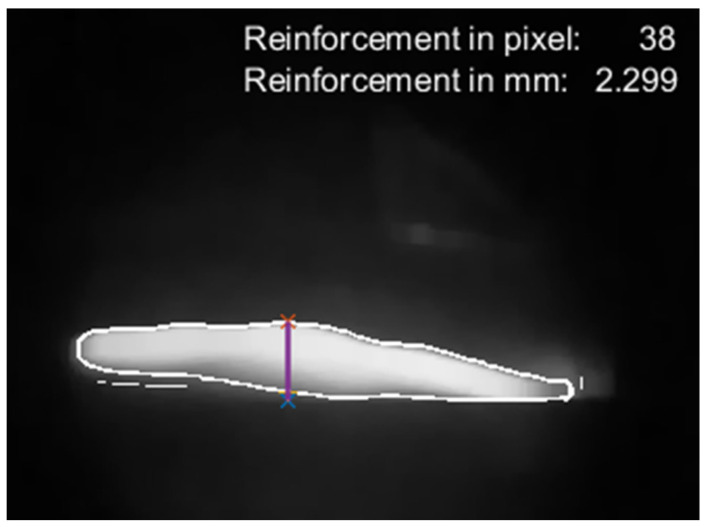
Final image illustrating reinforcement measurement, with pixel and millimeter scales.

Figure 8 illustrates the final result generated by the algorithm, displaying the reinforcement values in both pixels and millimeters. Additionally, the figure shows a line marking the exact point where the reinforcement is being measured.

#### 3.2.3. Dynamic Positioning of the Reinforcement Through the Measurement of the Crater Length (Dc)

**Figure 9:**
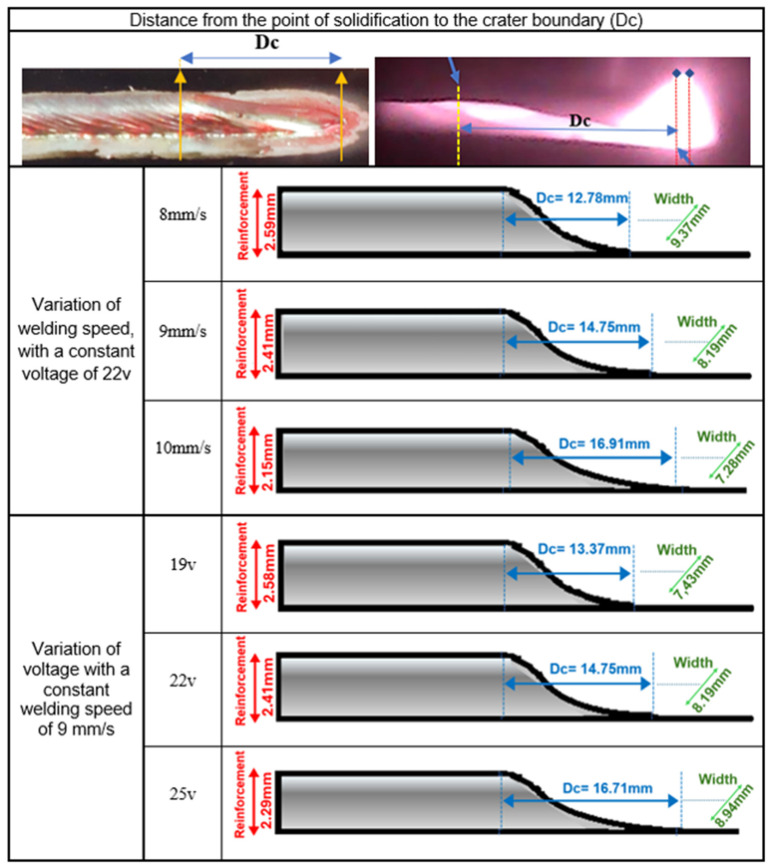
A summary of the relationship between welding speed, electrical arc voltage, and weld pool crater length (Dc).

**Figure 10:**
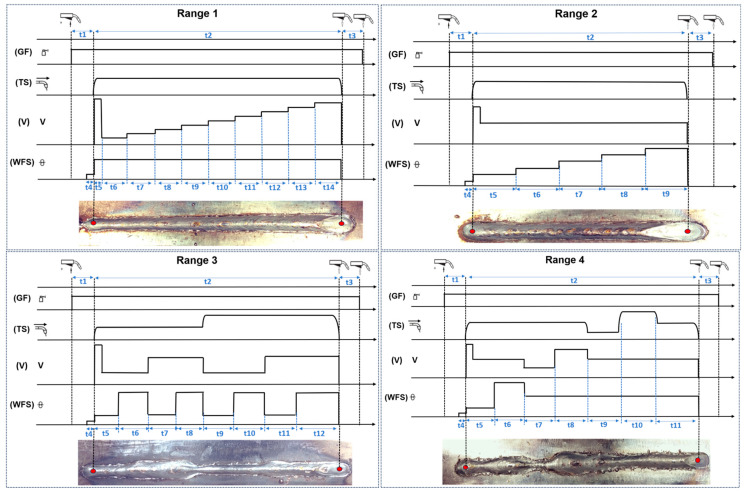
Diagram of the welding parameters corresponding to the four ranges applied in the experimental procedure: Range 1—proportional increase in arc voltage; Range 2—proportional increase in wire feed speed; Range 3—simultaneous average variation in three variables; Range 4—Simultaneous variation in three variables.

The relationship expressed in Equation (1) was derived from experimental data, correlating the crater length (Dc) with variations in welding speed (Ws) and arc voltage (V). Wire feed speed (Wfs) and welding power (P) were also considered, with P being indirectly represented through voltage variations. The crater length, denoted by Dc in Figure 9, can be estimated through a simplified linear regression model based on the variables Ts and V, as shown in Equation (1). The weld bead crater was used, along with the recordings of the molten pool, to estimate the molten pool length. In these tests, the arc termination parameters were kept constant to avoid any direct influence on the crater geometry. The details of the experimentally applied parameters and the diagram showing the corresponding values are presented in Table 3 and Figure 10. These allow visualization of the four parameters’ applied ranges and their effects on the resulting weld beads.

Dc = C_1_·Ws + C_2_·V + C_3_ (1)

where:

- Dc is the crater length (mm), or the distance from the wire to the measurement point.
- Ws is the welding speed (mm/s).
- V is the arc voltage (V).
- C_1_, C_2_, and C_3_ are empirical regression coefficients determined experimentally.

This regression model was established from 35 test cases. When the plasma, arc length, and part of the weld bead are visible in the image, the measurement point for the reinforcement must be adjusted accordingly, using the regression equation to improve accuracy.

**Table 3:** Abbreviations and symbols employed in the range-based description of welding parameters of Figure 10.

Variable	Abbreviations	Symbol
Gas Flow	(GF)	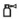
Travel Speed	(TS)	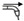
Voltage	(V)	V
Wire Feed Speed	(WFS)	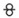
Time Per Section	(t)	

The parameters C_1_ and C_2_ and the constant C_3_ were determined by linear regression, resulting in

Dc = 2.065 Ws + 0.5567·V − 15.9537(2)

It is important to note that this equation is only applicable to the specific materials and parameters used in the analysis. Changes in filler wire type, shielding gas, or filler metal transfer mode may require adjustments to the equation parameters.

#### 3.2.4. Analysis Under Critical Conditions of Low Power or Start of Welding

**Figure 11:**
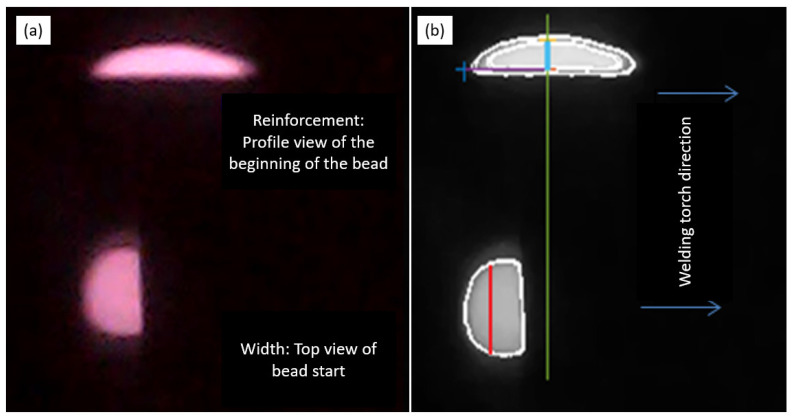
Measuring the weld bead geometry at the beginning of its formation for a test with critical parameters—low-power. (**a**) Images displaying the bead profile (reinforcement) and top view (width). (**b**) Result of applying an edge algorithm to the images to determine reinforcement height and width. The red and blue lines indicate the width and reinforcement measurements obtained by the image processing algorithm, while the remaining lines serve as references for identifying the images captured by the camera.

For tests involving minimal weld bead reinforcement, a weak electric arc, or the beginning of a weld bead, which typically presents a different shape, the algorithm is still capable of performing measurements with minimal error when compared to experimental data. Figure 11a,b show the top and side views of the beginning of a weld bead formation, along with the image segmentation performed by the algorithm. The image matrix is divided to allow for separate and precise measurements of both bead width and reinforcement.

#### 3.2.5. Bead Width Estimation Based on Molten Pool Geometry and Camera Angle Correction

To determine the weld bead width, a common approach involves measuring the distance between the outer edges of the bead. This measurement can be taken in the same column of the image where the reinforcement is evaluated.

Alternatively, this study proposes estimating the weld bead width based on the weld pool width. To model the relationship between the weld pool width and the final weld bead width, Equation (3) was formulated. This equation incorporates the influence of the wire feed speed (Wfs) and the welding speed (Ws), recognizing that these parameters impact the heat input and molten metal behavior. Specifically, the downward flow of liquid metal during solidification—driven by gravity—can lead to an increase in the final weld bead width compared to the initial weld pool width.

(3) $$Bw=Pw\times \left(1+\frac{0.01\times Ws}{Ts}\right)$$

Equation (3) is valid for the following parameter ranges:

- 0.2 ≤ Ws ≤ 0.9 m/min;
- 3 ≤ Wfs ≤ 12 m/min;

where:

- Bw is the predicted weld bead width (mm);
- Pw is the weld pool width (mm);
- Ws is the welding speed (mm/s);
- Wfs is the wire feed speed (mm/s).

This model allows for real-time estimation of weld bead geometry based on process parameters and vision system measurements.

**Figure 12:**
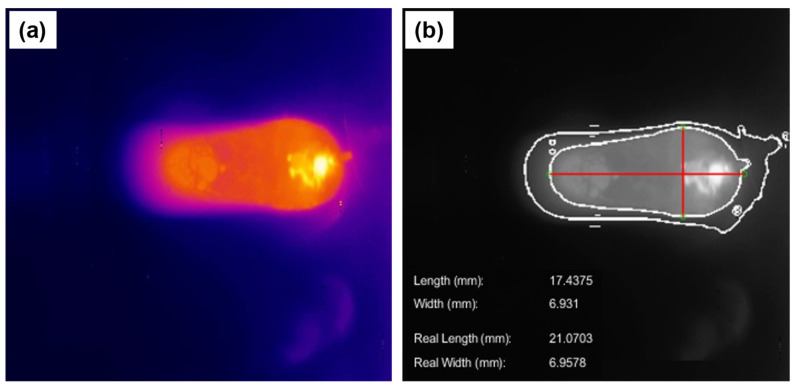
Weld pool length and width measurements. (**a**) Top view of the weld bead (image sector 2). (**b**) Segmentation and camera angle correction for weld bead and weld pool analysis with annotations showing the measured dimensions of weld pool width and length.

Figure 12 illustrates the segmentation of the weld pool, emphasizing the delineation of the solid–liquid interface within the molten region. This interface is inferred based on the emissivity data acquired via an infrared camera, corresponding to the known melting temperature of the base metal. Pixel intensity values, ranging from 1 to the maximum value of 255, are converted into temperature estimates by applying a fourth root transformation, given that radiative emissivity is governed by the Stefan–Boltzmann law, which relates emissive power to the fourth power of temperature. Following this, the geometric dimensions of the weld pool—namely, its width and length—are extracted in pixel units and subsequently converted into metric units (millimeters) using a pre-established calibration factor derived from the camera’s fixed working distance. To ensure spatial accuracy, a geometric correction is applied based on the angular offsets α and β between the camera’s optical axis and the normal to the deposition plane. The corrected measurements, denoted as ‘actual width’ and ‘actual length’, are presented in Figure 12b.

In conclusion, the proposed vision system enables real-time, non-invasive, and low-cost monitoring of weld bead geometry. By carefully selecting filters and tuning the camera, high-quality IR images were obtained in the 1000–1150 nm band. The system accurately measures weld bead reinforcement and width, as well as weld crater length, key parameters for quality assurance in GMAW.

### 3.3. Vision System Data Processing

A real-time acquisition system integrated with LabVIEW enabled the synchronized collection of signals from various sensors—specifically current, electric arc voltage, wire feed speed, and infrared vision system images—during welding. This setup allowed dynamic process monitoring, while MATLAB^®^ was used for subsequent data analysis, including refined processing, segmentation, and quantitative evaluation.

**Figure 13:**
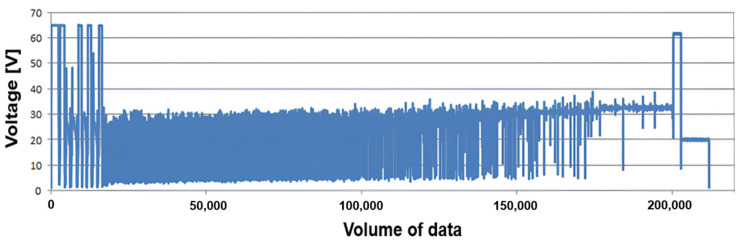
Short-circuit transfer mode voltage oscillogram.

In the context of the 35 welding trials, data were collected using two different experimental methodologies: the first based on a central composite design, which provides statistically representative variation, and the second using a parameter sweep method, where one variable is altered at a time while the others remain constant. The first 10 results are cataloged in Table 4, where it is possible to see a fluctuation in the results due to the instability in the first milliseconds due to the arc opening time so that the process stabilizes after 1 s. This variation in the electrical signal at the moment of arc opening and subsequent stabilization can be seen in detail in Figure 13, where the oscillogram of the short-circuit transfer mode is displayed. This algorithm is capable of extracting key features from each image, providing accurate data on the weld bead dimensions, such as reinforcement and bead width, as well as generating a graphical profile of the reinforcement line, along with measurements of the weld pool width and electric arc length.

In total, for each weld bead, between 1000 and 2000 data points were collected at a frequency of 30 Hz, with each record including information on both input and output parameters—such as weld bead width and reinforcement and electric arc length. For the final analysis, only four weld beads were generated, summarizing the variations from the 35 tests. The first weld bead represents a voltage sweep with all other process parameters held constant. The second weld bead corresponds to a wire feed speed sweep under fixed conditions for the remaining variables. The final two weld beads each encompass a broad range of input parameter variations within a single weld pass, as defined by the experimental runs in the central composite design.

**Figure 14:**
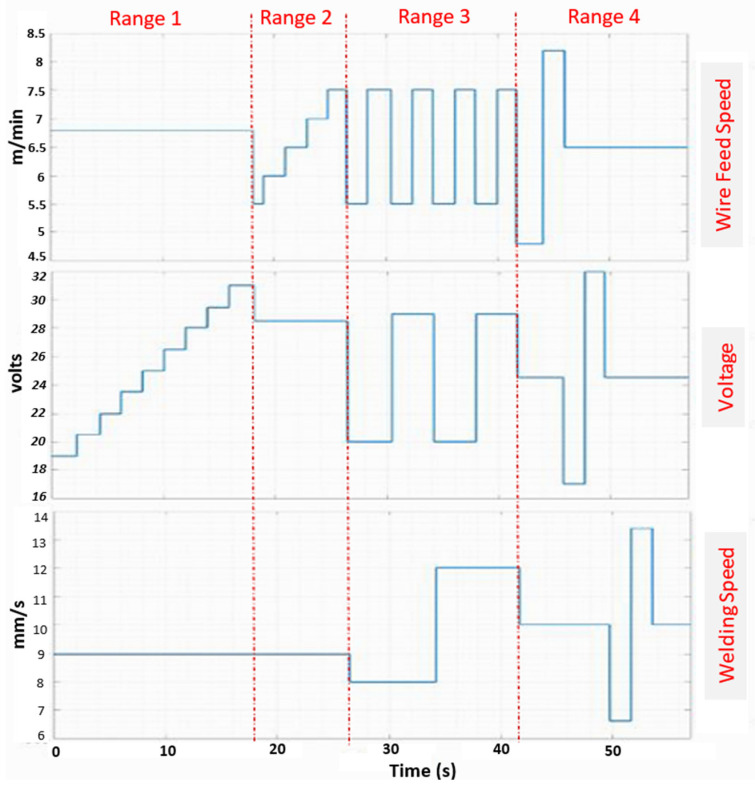
Temporal variation of three input variables for the power source and robot controllers.

Figure 14 illustrates how the three input parameters (Wfs, Ws, and V) vary throughout the tests. These variations are separated by red dashed lines, each marking the start and end of a weld bead, resulting in four distinct beads. Excluding the first and last 300 milliseconds of each bead, a total of 6800 data points were collected during the welding of the four beads. These correspond to 6800 values for weld bead reinforcement and width, weld pool length, and electric arc length, acquired in real time.

This extensive dataset provides a solid foundation for statistical and trend analyses, which are crucial for a deeper understanding of welding and for future work aiming to develop predictive models using neural networks and more effective control strategies. The combined use of LabVIEW and MATLAB^®^ in this context highlights the importance of software tools in modern engineering for the optimization and control of complex industrial processes such as welding.

The algorithm was capable of processing images every 10 milliseconds to calculate the reinforcement (height) and width of the weld bead, using segmentation with enhanced contrast and edge detection. This is important because the obtained images were captured every 33 milliseconds, and the current and electric arc voltage data were captured every 10 milliseconds. Therefore, the algorithm must process faster than the image is generated and repeat the data three times to synchronize the measurement outputs related to the image with the current and voltage values. The measurements were also corrected through adjustments and calibration, with a fit performed for each test based on the correlation between pixels and millimeters, as well as the necessary compensation due to mirror distortions and camera positioning.

**Figure 15:**
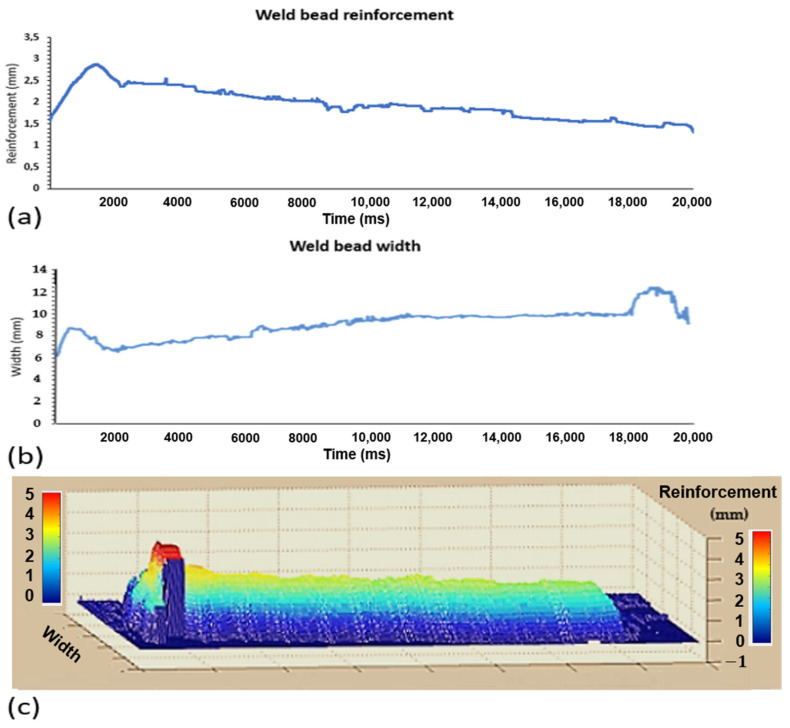
Temporal evolution of the weld bead reinforcement and width: (**a**) reinforcement data, (**b**) width data, and (**c**) from real-time infrared image analysis (MATLAB R2016b, with 3D laser scan validation). Color scale indicates pixel intensity variation: vertical changes correspond to height differences, while horizontal changes reflect increasing surface planarity.

To evaluate the performance of the vision system, time series graphs were generated to determine the weld bead reinforcement and width. Figure 15 shows the weld bead test where only the electric arc voltage is varied using the sweep method. A total of 2000 data points were collected, varying the electric arc voltage nine times in an increasing manner:

A controlled variation of input variables was implemented to investigate the relationship between welding parameters and weld bead geometry. The power source and the robot controller were programmed to vary electric arc voltage, wire feed speed, and welding speed following defined patterns.

- Range 1: gradual increase in electric arc voltage;
- Range 2: gradual increase in wire feed speed;
- Range 3: alternating variations in all parameters;
- Range 4: full-range random variation in the three variables.

Visual inspection of the resulting weld beads (Figure 10) reveals distinct differences in weld bead consistency and shape across each range. These observations directly correlate with the geometric variations measured by the vision system, providing qualitative validation of the automated measurements.

**Figure 16:**
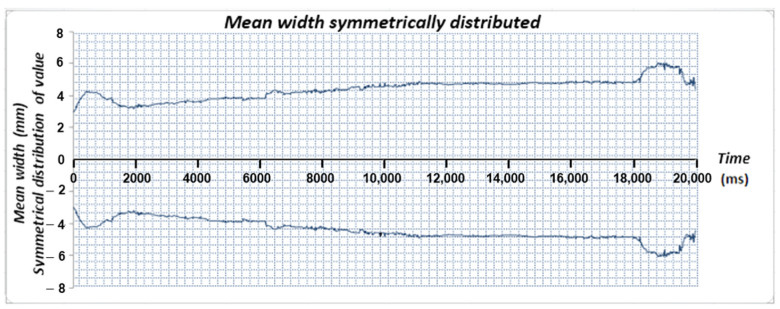
Dispersion of the data collected from Figure 15b to the left and right of the mean value, to visualize the actual shape of the weld bead viewed from the top.

To improve the interpretation of the weld bead shape, as shown previously, the time-series data of the weld bead width from Figure 15b were symmetrically mirrored around the mean width value. This approach, illustrated in Figure 16, reconstructs the top-view profile of the weld bead, highlighting areas of asymmetry and dimensional variation. Figure 16 demonstrates how real-time image data can better approximate the actual weld bead geometry.

The integration of software, sensor hardware, and computer vision enabled precise real-time quantification of weld bead reinforcement and width, facilitating robust welding monitoring. The system’s ability to capture rapid geometrical changes validates its suitability for online control applications. Furthermore, the agreement between real-time results and offline scanning demonstrates that low-cost vision-based monitoring can effectively replace more complex and expensive techniques, offering a scalable solution for industrial welding automation.

### 3.4. Camera Calibration

Camera calibration is fundamental to ensuring the accuracy and reliability of measurements obtained from images. It establishes a precise relationship between image pixels and real-world metric units (typically millimeters), which is essential for dimensional analysis in computer vision.

Several calibration techniques are bellow. Prominent methods include the following:

- Direct linear transformation (DLT) is known for its simplicity and ease of implementation. However, it does not account for lens distortions, significantly limiting its precision in applications requiring geometric fidelity.
- The MATLAB R2016b Camera Calibration Toolbox is a widely used toolbox offering a robust solution that includes lens distortion correction. Its ease of use and precision make it suitable for most engineering and scientific applications demanding accurate camera calibration.
- Tsai’s method [29] is a more sophisticated model that accounts for lens distortions and nonlinearities. Although more complex to implement, this method offers high accuracy and was adopted in this study. It was chosen based on previous work by the authors using a CMOS Lumenera LW230 camera (1616 × 1216 pixels, 4.4 μm pixel pitch), which demonstrated its suitability for precise dimensional applications in computer vision.

The intrinsic parameters obtained from Tsai’s calibration for this setup are presented in Table 5:

These parameters were used throughout the experiments to correct geometric distortions and convert pixel measurements to metric units.

Furthermore, synchronized measurement of multiple parameters was achieved through the integration of the vision system with electrical sensors and data acquisition software, resulting in a richer and more reliable dataset. The accuracy of the image processing algorithms was validated using laser profilometry and manual caliper measurements, confirming the system’s reliability in geometric assessment. Camera calibration using Tsai’s method was instrumental in ensuring consistent and repeatable measurements across all experimental conditions.

### 3.5. Integration with Machine Learning Application

The experimental procedures and data processing techniques detailed in this study yielded a comprehensive and consistent dataset, subsequently used to train a Support Vector Machine (SVM). Furthermore, the data organization and preprocessing methodology were based on the work of Park et al. [30] and Xiao et al. [31]. These references significantly informed the dataset structuring and the exploration of correlations among multiple nonlinear parameters, especially under dynamic or chaotic system behaviors.

These insights motivated the development of a Machine Learning architecture specifically designed to model welding behavior under real-world variability. Furthermore, this study suggests using Support Vector Machine (SVM) models to classify and predict welding outcomes based on vision and sensor data. The robust framework established in this study supports real-time process control and highlights the potential of integrating vision systems with machine learning for intelligent manufacturing applications.

For machine learning SVMs, the Gaussian function was chosen for the kernel (K), which is the network activation function for each vector or neuron, and the mean squared error (MSE) was used to relate the errors caused by noise. Training was performed for each desired output (width, reinforcement, and penetration). The developed program allows choosing between multiple possible models generated with different neurons, which may have other errors, different stabilization times, and increased or decreased complexity of the SVM structure. The program performs 100 different training exercises using the same data for 100 distinct sets of neurons. In the end, the model selects the most suitable data for the smallest number of neurons, thereby avoiding overfitting and preserving the tool’s generalization capability.

**Figure 17:**
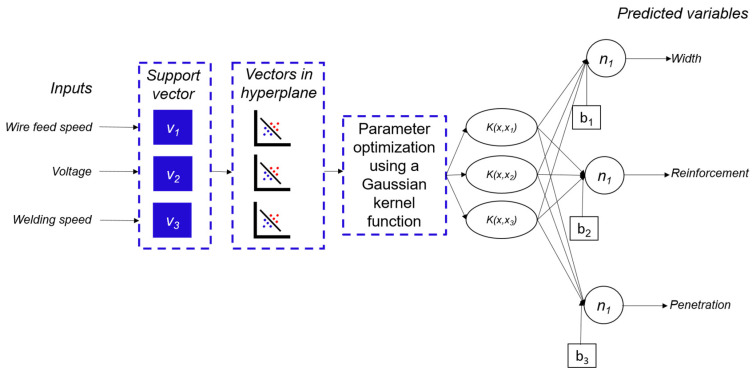
SVM architecture.

The input data for the developed tool are wire feed speed, open-circuit voltage, and nominal welding speed. The output data are weld bead width, reinforcement, and penetration. It is essential to note that the classification model was not directly trained on the images captured by the cameras. Instead, the image processing algorithm described earlier was used to extract measurements of the molten pool geometry, which were then employed in training the SVM. Figure 17 provides a detailed overview of the model’s architecture.

**Figure 18:**
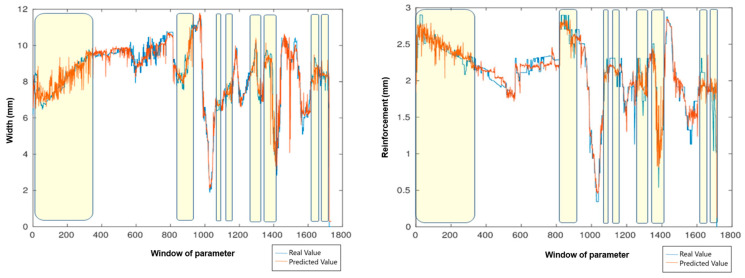
Comparison between predicted and real values of the weld joint profile. The yellow regions indicate the short-circuit transfer mode.

The result of the comparisons between the real values and the values estimated by the SVM prediction tool is shown in Figure 18, where the blue value is the real value for width and reinforcement, and the red value is the value estimated by the SVM. The yellow boxes are the areas where the short-circuit transfer mode is present. Although it is a transfer mode that is difficult to predict due to its instability and abruptness, the model achieved good generalization in the prediction tool. A key point to note is that the experimental measurements, obtained via profilometer and caliper, correspond to the width and reinforcement, with a standard deviation of approximately 0.001.

Using infrared image capture instruments, it was possible to monitor the different transfer modes, as well as the points near these changes, detecting possible failures or the generation of spatter during welding. The CCD camera was also likely to evaluate the change in the geometry of the weld bead in real time during the welding process. Thus, an online monitoring system was developed, and by coupling it to the SVM, a system for predicting the geometry and transfer mode was created, capable of detecting anomalies in the geometry of the weld bead based on the electrical parameters and the transfer mode of the drop. Given that the trained tool synchronizes the electrical signals with the bead geometry extracted using the image processing algorithm outlined above, it is possible to correlate them with the instantaneous values of wire feed speed, welding speed, and voltage.

In addition to the geometry monitoring system, the tool also plays a significant role when applied as a predictive tool for weld bead geometry. This enables the calculation of overlap between beads and layers in an additive manufacturing context, eliminating the need for profilometers and calipers during part printing. Consequently, the printing process is optimized by establishing an effective correlation between welding parameters and the final geometry.

## 4. Conclusions

This study demonstrated the feasibility of implementing a low-cost, real-time vision system for the analysis of weld bead geometry and measurement of the weld pool in Gas Metal Arc Welding (GMAW). A key contribution was the investigation of the spectral behavior of argon and CO_2_ gas mixtures, which enabled the optimization of infrared imaging in the (1000–1150 nm) range. This spectral window allowed for high-quality image acquisition without the need for external illumination sources. The proposed system successfully performed real-time measurements of weld bead reinforcement and width, as well as weld pool length and width, even in challenging low-power welding scenarios. These findings support the system’s potential as a practical and accessible solution for monitoring and controlling welding processes with high precision.

The resulting dataset proved effective for training the SVM model, highlighting the system’s potential for predictive modeling and intelligent control in welding applications. The system’s sub-millimeter accuracy in measuring and analyzing weld bead geometry, achieved with accessible and affordable technology, represents a significant advancement in automated welding inspection.

In conclusion, this work highlights the synergistic integration of optics, signal processing, and machine learning, and it validates the use of embedded vision systems as practical tools for quality assurance and control in modern manufacturing environments.

## Figures and Tables

**Table 1:** Chemical composition of wires (weight%).

Wire	C	Si	Mn	Cr	Ni	Mo	N
AISI 316L	0.015	0.45	1.6	18.5	12.0	2.6	0.04
E410NiMoT1-4	0.05	0.50	0.30	13.0	4.0	0.55	<0.01

**Table 2:** Emission spectra of commercial LEDs.

Wavelength (nm)	Color
380–450	Violet
450–490	Blue
490–520	Cyan
520–570	Green
570–590	Yellow
590–620	Orange
620–740	Red

**Table 4:** Sample of the first 10 data points (a total of 6800 data points) for geometry analysis and validation of the weld beads obtained through the developed algorithm and camera.

AWS NiMo Wire—Ar—4% CO_2_ Shielding Gas						
Data	Time [ms]	Wire Feed Speed [m/min]	Welding Speed [mm/s]	Open Loop Voltage [V]	Current [A]	Arc Voltage [V]
1	10	6.80	9	19	264.23	22.44
2	20	6.80	9	19	337.85	5.40
3	30	6.80	9	19	96.65	17.37
4	40	6.80	9	19	621.00	10.66
5	50	6.80	9	19	135.64	22.26
6	60	6.80	9	19	74.41	17.60
7	70	6.80	9	19	76.62	16.45
8	80	6.80	9	19	270.31	24.67
9	90	6.80	9	19	329.99	5.80
10	100	6.80	9	19	104.85	18.08
**Data**	**Arc length [mm]**	**Stick out [mm]**	**Weld bead Width [mm]**	**Weld bead reinforcement [mm]**	**Penetration [mm]**	**Transfer mode**
1	1.38	13.62	6.01	1.72	0.69	CC-(1)
2	1.38	13.62	6.01	1.72	1.14	CC-(1)
3	1.385	13.62	6.01	1.72	1.09	CC-(1)
4	1.50	13.50	6.19	1.92	1.05	CC-(1)
5	1.50	13.50	6.19	1.92	0.94	CC-(1)
6	1.50	13.50	6.19	1.92	0.96	CC-(1)
7	1.50	13.50	6.36	1.92	0.96	CC-(1)
8	1.38	13.62	6.36	1.92	0.96	CC-(1)
9	1.38	13.62	6.36	1.92	0.96	CC-(1)
10	1.38	13.62	6.77	2.11	0.96	CC-(1)

**Table 5:** Intrinsic parameters of the calibrated camera.

Focal Length *(f)* (mm)	Image Center *(Cx, Cy)* (Pixels)	Scale Factors *(sx, sy)* (Pixels/mm)	Radial Distortion Coefficient *(k)*
9.43773	(738, 585)	(227.27, 227.27)	9.4604 × 10^−9^

## Data Availability

The original contributions presented in this study are included in the article. Further inquiries can be directed to the corresponding author(s).

## Appendix A

**Algorithm A1** Image processing algorithm
Step 1 NumF= input (‘Write the number of frames you will analyze. NumF: ’); For a=1:NumF %This for allows analyzing several frames filename = [“Img”,num2str(a,‘R’),”.jpg’]; route = [‘C:\Program Files \MATLAB\R2016a\bin\img\’filename]; Img1 = imread(route); %APPLICATION OF MEDIA FILTER AND CHANGE TO GRAY TONES Img2 = uint8(Img); for j = 5: NumF-5 for i = 7: NumF/4-7 Img3(i,j) = median((Img2(i − 6:i + 6,j − 4:j + 4))); end end Gray = rgbgray(Img3); Step 2 % Binarization % Upper edge binarization—External heat radiation Edge1 = edge(Gray ≤ 100,‘sobel’); Edge2 = edge(Gray ≤ 100,‘log’); Edge3 = edge(Gray ≤ 100,‘canny’); EdgeF = imadjust(Edge1 + Edge2+ Edge3); Step 3 Edge12 = edge(gray ≤ 200,‘sobel’); Edge22 = edge(Gray ≤ 200,‘log’); Edge32 = edge(Gray ≤ 200,‘canny’); EdgeF2 = imadjust(Edge12 + Edge22 + Edge32); Step 4 Xm= average(midpoint_external, midpoint_internal); XR= round(Xm × 0.95); Step 5 Reinforcement= End_Reinforcement—Start_Reinforcement; Reinforcement_mm= Reinforcement × 0.0605; %Pixel to mm, conversion Factor.
